# Toll-Like Receptor 4 Is Essential in the Development of Abdominal Aortic Aneurysm

**DOI:** 10.1371/journal.pone.0146565

**Published:** 2016-01-07

**Authors:** Chao-Han Lai, Kuan-Chieh Wang, Fang-Tzu Lee, Hung-Wen Tsai, Chih-Yuan Ma, Tsung-Lin Cheng, Bi-Ing Chang, Yu-Jen Yang, Guey-Yueh Shi, Hua-Lin Wu

**Affiliations:** 1 Department of Surgery, National Cheng Kung University Hospital, College of Medicine, National Cheng Kung University, Tainan, Taiwan; 2 Cardiovascular Research Center, College of Medicine, National Cheng Kung University, Tainan, Taiwan; 3 Department of Biochemistry and Molecular Biology, College of Medicine, National Cheng Kung University, Tainan, Taiwan; 4 Department of Pathology, National Cheng Kung University Hospital, College of Medicine, National Cheng Kung University, Tainan, Taiwan; 5 Department of Physiology, College of Medicine, Kaohsiung Medical University, Kaohsiung, Taiwan; Stellenbosch University Faculty of Medicine and Health Sciences, SOUTH AFRICA

## Abstract

Toll-like receptor (TLR) family plays a key role in innate immunity and various inflammatory responses. TLR4, one of the well-characterized pattern-recognition receptors, can be activated by endogenous damage-associated molecular pattern molecules such as high mobility group box 1 (HMGB1) to sustain sterile inflammation. Evidence suggested that blockade of TLR4 signaling may confer protection against abdominal aortic aneurysm (AAA). Herein we aimed to obtain further insight into the mechanism by which TLR4 might promote aneurysm formation. Characterization of the CaCl_2_-induced AAA model in mice revealed that upregulation of TLR4 expression, localized predominantly to vascular smooth muscle cells (VSMCs), was followed by a late decline during a 28-day period of AAA development. *In vitro*, TLR4 expression was increased in VSMCs treated with HMGB1. Knockdown of TLR4 by siRNA attenuated HMGB1-enhanced production of proinflammatory cytokines, specifically interleukin-6 and monocyte chemoattractant protein-1 (MCP-1), and matrix-degrading matrix metalloproteinase (MMP)-2 from VSMCs. *In vivo*, two different strains of TLR4-deficient (C57BL/10ScNJ and C3H/HeJ) mice were resistant to CaCl_2_-induced AAA formation compared to their respective controls (C57BL/10ScSnJ and C3H/HeN). Knockout of TLR4 reduced interleukin-6 and MCP-1 levels and HMGB1 expression, attenuated macrophage accumulation, and eventually suppressed MMP production, elastin destruction and VSMC loss. Finally, human AAA exhibited higher TLR4 expression that was localized to VSMCs. These data suggest that TLR4 signaling contributes to AAA formation by promoting a proinflammatory status of VSMCs and by inducing proteinase release from VSMCs during aneurysm initiation and development.

## Introduction

Toll-like receptors (TLRs) are type I transmembrane proteins that play a key role in innate immunity and various inflammatory responses. Activation of TLRs and downstream signaling has important physiological, immunological and pathological significance [1–6]. TLR4, one of the most extensively characterized pattern-recognition receptors (PRRs), is the receptor for lipopolysaccharide derived from gram-negative bacteria. TLR4 can also be activated by endogenous damage-associated molecular pattern molecules (DAMPs, e.g., high mobility group box 1 [HMGB1] and S100 family) [2,3,6,7]. Endogenous ligand-TLR4 interaction may initiate positive feedback loops where increasing tissue damage perpetuates proinflammatory responses [3,8]. Recent findings have revealed a previously unappreciated involvement of TLR4 in sterile inflammation among a variety of cardiovascular diseases such as myocardial ischemia-reperfusion injury and stroke [1,2,4,5,9–11].

Abdominal aortic aneurysm (AAA), a common vascular disease in the elderly population, is a degenerative process of the abdominal aorta [12]. Clinically, AAAs can be repaired using open surgery and endovascular technique. However, the two established therapeutic managements are only indicated for patients whose AAA has surpassed 5.5 centimeters in diameter, i.e. a size with a substantially increased risk of lethal rupture [13,14]. Nowadays physicians remain incapable of modifying the natural history of AAA progression. Nevertheless, more and more studies aimed at understanding the underlying events that support AAA development and developing potential therapeutic strategies that modify the disease course of AAA [13,15]. Over the past 2 decades, researchers have begun to understand the molecular mechanisms driving aneurysm formation through studies of human specimens and three mouse AAA models (i.e., angiotensin II [AngII]-infusion model, CaCl_2_-induced model, and elastase-perfusion model). The three established mouse AAA models not only serve as a platform to perform pre-clinical studies for potential drugs but also help gain mechanistic insight into the sequence of biological processes during AAA development [13,15,16]. Sustained inflammation, as represented by upregulation of proinflammatory cytokines (e.g., tumor necrosis factor-α [TNF-α], interleukin-6 [IL-6] and monocyte chemoattractant protein-1 [MCP-1]) and infiltration of inflammatory cells (e.g., macrophages), may play a fundamental role during the initiation and development of AAA. In addition, increased production of matrix-degrading proteinases (e.g., matrix metalloproteinases [MMPs] and cysteinyl cathepsins) may result in destruction of elastin, loss of vascular smooth muscle cells (VSMCs) and consequent weakening of the aortic wall [13,16–18]. Signaling pathways that underpin vascular inflammation and proteolysis of the aortic vasculature in these models may serve as therapeutic targets for potential pharmacotherapies of AAA [13,14,16].

Studies have shown that HMGB1 and S100A12, another recently recognized TLR4 ligand [19], contribute to vascular inflammation and MMP production, implicating a role of TLR4 in aneurysm lesions [12,20,21]. In addition, in one study focusing on the role of MyD88 in AAA, TLR4 deficiency attenuates AngII-infusion AAA in low-density lipoprotein receptor (LDLR)-deficient mice [22]. Also, Tanshinone IIA, a major extract from the traditional Chinese herb Salvia miltiorrhiza, inhibits elastase-induced AAA in rats possibly via inhibition of TLR4 signaling [23]. Despite these findings, the mechanism by which TLR4 might positively regulate aneurysm formation remains mostly unclear. In studies reported here, we have investigated the hypothesis that TLR4 might be essential in the pathogenesis of AAA. Using the CaCl_2_-induced AAA model in mice, TLR4 expression was evaluated during AAA formation. The effects of TLR4 inhibition by siRNA on cytokine and MMP production were explored *in vitro*. The experimental AAA model was induced in two strains of TLR4-deficient mice versus their respective controls to appraise the *in vivo* responses of TLR4 signaling blockade in AAA. Finally, human specimens were inspected for the potential clinical relevance of TLR4. These studies would help elucidate the role that TLR4 might play in AAA.

## Materials and Methods

### Mouse AAA model

The AAA model was induced by CaCl_2_ in male mice at age of 8 to 10 weeks as described previously [12,24,25]. After premedication with atropine, a laparotomy was performed under general anesthesia using intraperitoneal injection of sodium pentobarbital. The adequacy of anesthesia was monitored by loss of tail pinch and pedal withdrawal reflexes. After the abdominal aorta was exposed, a 26-gauge needle (0.45 mm in diameter) was placed parallel to the aorta, and the image was acquired with a digital camera attached to Zeiss dissection microscope. Subsequently, a small piece of cosmetic cotton pad soaked in 0.5 M CaCl_2_ solution was applied periaortically for 15 minutes. In sham group mice, CaCl_2_ was replaced with 0.9% NaCl. At indicated time points, the mice were sacrificed by phenobarbital overdose for image acquisition. The infra-renal aorta of each mouse was harvested for further biochemical and histological analysis. The point of the maximal aortic diameter was preferentially measured in proportion to the needle diameter under ImageJ software.

For TLR4 expression during AAA development, C57BL/6 mice that do not carry any genetic manipulation were used. To investigate the *in vivo* effects of TLR4 in AAA, C57BL/10ScNJ (TLR4-knockout; stock number: 003752) and C57BL/10ScSnJ (TLR4 wild-type; stock number: 000476) mice purchased from The Jackson Laboratory (Bar Harbor, ME), C3H/HeJ (TLR4-mutant) mice from CLEA Japan (Tokyo, Japan) and C3H/HeN (TLR4 wild-type) mice from Charles River Laboratories Japan (Yokohama, Japan) were used. Both C57BL/10ScNJ and C3H/HeJ mice do not express functional TLR4. A deletion of the TLR4 gene was represented in C57BL/10ScNJ mice, and a single point mutation in the TLR4 gene in C3H/HeJ mice. C57BL/10ScSnJ and C3H/HeN mice that do not express the mutation are considered to be their respective controls. The experimental designs conform to the *Guide for the Care and Use of Laboratory Animals* published by the National Institutes of Health (NIH Publication #85–23, revised 1996), and this study were approved by Institutional Animal Care and Use Committee of National Cheng Kung University (approval number: 101117).

### Quantification for TLR4, receptor for advanced glycation end-product (RAGE) and HMGB1 in mouse aortas or cell lysates

The levels of TLR4, RAGE and HMGB1 in mouse aortas or cell lysates were analyzed using Western blot analysis. Equal protein amounts of mouse aortic homogenates were resolved on 10% sodium dodecyl sulfate-polyacrylamide gel electrophoresis gels and were transferred to nitrocellulose by electroblotting. The membrane was blocked with PBS and Tween 20 containing 5% non-fat dry milk to prevent nonspecific antibody binding. After being probed with appropriate dilution of antibodies against TLR4 (Abcam, Cambridge, MA; catalog number: ab13556; rabbit polyclonal antibody), RAGE (H-300; Santa Cruz Biotechnology, Santa Cruz, CA; catalog number: sc-5563; rabbit polyclonal antibody) and HMGB1 (Abcam; catalog number: ab18256; rabbit polyclonal antibody) overnight at 4°C, the membrane was incubated with a horseradish peroxidase-conjugated secondary antibody for 1 hour at room temperature. The signal was detected using an enhanced chemiluminescence reagent (Amersham Pharmacia Biotech, Piscataway, NJ). The band intensity was quantitatively measured using ImageJ software. β-actin (Sigma-Aldrich, St Louis, MO) or GADPH (Sigma-Aldrich) was used to confirm equal protein loading.

### Histological analysis of mouse aortas

Five-μm thick frozen sections of mouse aortas were applied for staining or immunostaining for TLR4 (1:100 dilution), macrophages (macrophage marker [MOMA-2], 1:200 dilution; Abcam; catalog number: ab33451; rat monoclonal antibody), VSMCs (α-smooth muscle actin [α-SMA], 1:400 dilution; Sigma-Aldrich; catalog number: A2547; mouse monoclonal antibody) and elastin degradation (Verhoeff-Van Gieson [VVG] staining). Isotype IgG control (Calbiochem, San Diego, CA) slides were provided as negative controls to confirm the specificity of respective primary antibodies in mouse AAA specimens (S6 Fig). All sections used for comparison were located at the maximal expansion of the infra-renal aorta and approximately at a similar distance from the renal arteries. These slides were visualized with a Carl Zeiss upright microscope. Digital images were acquired using a digital camera and were analyzed using ImageJ software. VSMC content, as represented by the intensity of α-SMA staining, was determined using HistoQuest software (TissueGnostics, Vienna, Austria), an automated image analysis system for quantification of protein expression [26]. Elastin fragmentation and macrophage counts were evaluated blindly by 2 independent observers. For each mouse, the intensity of α-SMA staining and numbers of elastin breaks and macrophages were measured in 3 serial 5-μm sections and the data were then averaged. The mean value from 6 mice in each group was presented.

### Quantification for proinflammatory mediators and MMPs in mouse aortas or cell culture supernatants

Equal amounts of mouse aortic homogenates or cell culture supernatants were analyzed using commercially available ELISA kits specific for mouse TNF-α (R&D systems, Minneapolis, MN), IL-6 (R&D systems), MCP-1 (R&D systems), total MMP-9 (Abnova, Taipei, Taiwan) and total MMP-2 (Abnova), and for human TNF-α (eBioscience, San Diego, CA), IL-6 (eBioscience), MCP-1 (eBioscience), total MMP-9 (R&D systems) and total MMP-2 (R&D systems) according to the manufacturers’ protocols. The protein concentration was determined by spectrophotometric optical density (450 nm) using an automated microplate reader (SpectraMax 340PC^384^; Molecular Devices, Sunnyvale, CA).

### Cell cultures

Human aortic smooth muscle cells (HASMCs; Cascade Biologics, Portland, OR) were maintained in medium 231 containing 5% smooth muscle growth supplement (SMGS) at 37°C in a humidified atmosphere of 5% CO_2_ and 95% air. HASMCs (between passages 3 and 5) were grown to 80–90% confluence and were made quiescent by serum starvation (0.1% SMGS) for 24 hours. Subsequently, HASMCs were cultured in fresh medium without stimulation or were treated with indicated concentrations of recombinant human HMGB1 (R&D systems) for 24 hours.

### SiRNA transfection

The cultured HASMCs were grown to 60–70% confluence and were transfected with equal amounts of TLR4 siRNA or control siRNA using DharmaFECT 2 (Thermo Fisher Scientific, Lafayette, CO) according to the manufacturer’s instructions. After 48-hour transfection, the cells were harvested. Western blot analysis and real-time polymerase chain reaction (PCR) were performed to determine the silencing effects of TLR4 siRNA.

### WST-1 viability assay

Cells viability in 96-well plates (5 ×10^3^/well) was assessed using the WST-1 assay according to manufacturer’s instructions (Roche, Mannheim, Germany). A 10-μL of WST-1 was added to the wells and was incubated for 1 hour. The absorbance was directly recorded at a wavelength of 450 nm in a microplate reader.

### Human aortic specimens

Human AAA specimens were obtained from 8 patients undergoing surgical repair of AAA. The paraffin-embedded tissue sections were retrieved from the tissue bank in National Cheng Kung University Hospital. Eight normal human aorta specimens obtained from autopsy (Pantomics, Richmond, CA; US Biomax, Rockville, MD; OriGene Technologies, Rockville, MD) were used for comparison. None of these individuals were known to have connective tissue disorders. This study was approved by the Institutional Review Board of National Cheng Kung University Hospital (approval number: A-ER-102-184). The informed consent was waived as the AAA specimens were de-identified. Immunohistochemistry with TLR4 antibody (1:50 dilution) was performed on 4-μm-thick formalin-fixed paraffin-embedded sections. The procedures were done with the Bond-Max automated immunohistochemistry stainer (Leica Biosystems, Melbourne, Australia). Counterstaining was carried out with hematoxylin. The intensity of TLR4 expression in the VSMCs was determined using HistoQuest software. To demonstrate possible co-localization of TLR4 and VSMCs or TLR4 and macrophages, AAA specimens were also applied for immunostaining for α-SMA (Sigma-Aldrich) and CD68 (1:50 dilution; DAKO, Carpinteria, CA; clone KP1; mouse monoclonal antibody), respectively. Isotype IgG control slides were provided as negative controls to confirm the specificity of respective primary antibodies in human AAA specimens (S6 Fig).

### Statistical analysis

Data were expressed as means ± SEM and statistical analyses were performed using Prism 5 (GraphPad Software; San Diego, CA). For comparisons between two groups, a Student’s *t*-test was used in data that passed both normality and equal variance; otherwise, a non-parametric Mann-Whitney U test was performed. For comparisons between multiple groups, one-way analysis of variance was used in data that passed both normality and equal variance, followed by post hoc analysis (Bonferroni test); otherwise, a nonparametric Kruskal-Wallis test was used, followed by post hoc analysis (Dunn test). A *P*<0.05 was considered statistically significant.

## Results

### Upregulation of TLR4 during experimental AAA formation in mice

**Fig 1:**
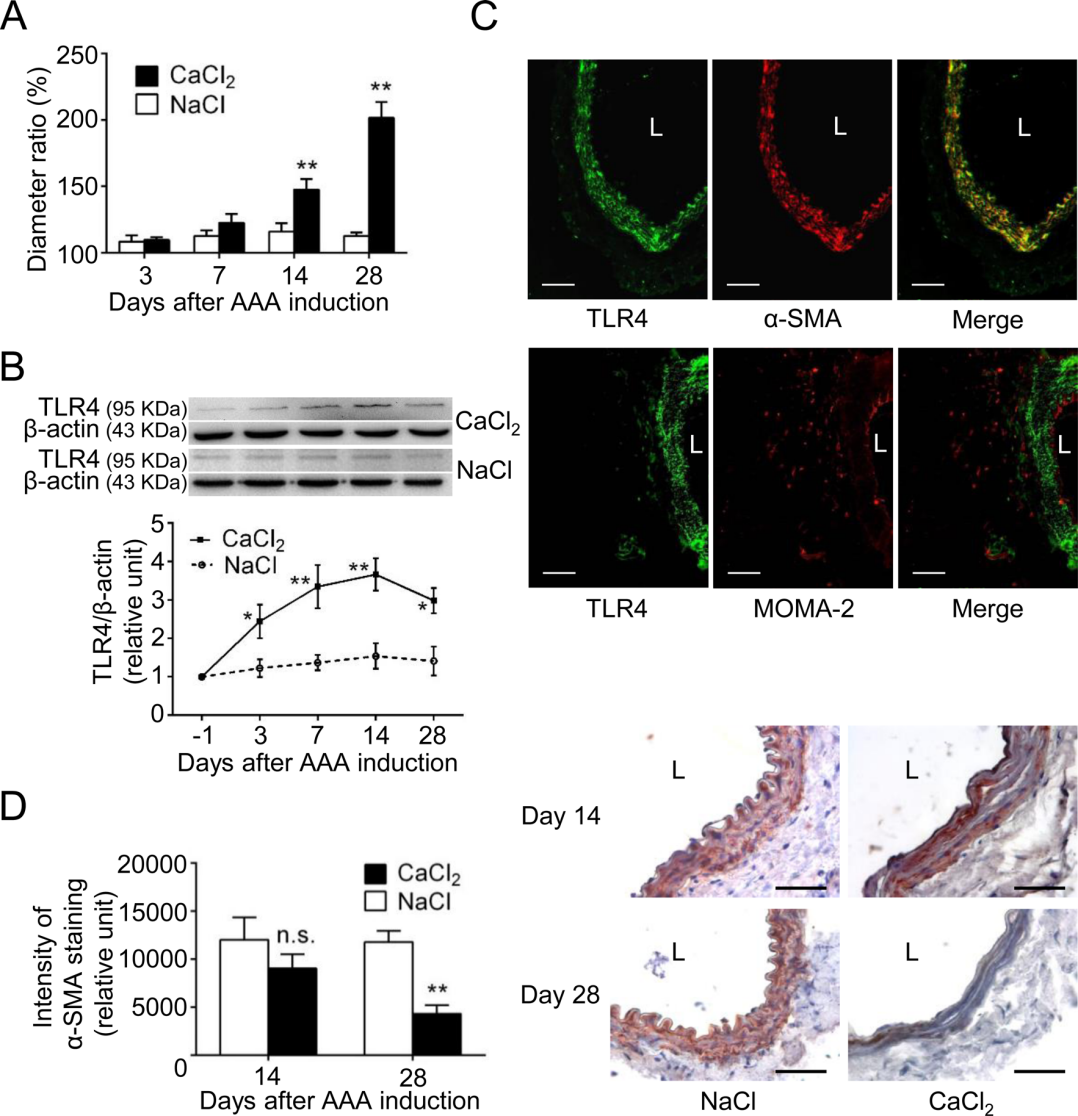
Upregulation of TLR4 during experimental AAA formation in mice. (A) Aortic diameter change during the 28 days of CaCl_2_-induced AAA formation (relative to day 0, n = 6 per group). (B) Time-course of TLR4 expression (relative to the level in normal aorta, n = 4 per group). (C) Representative microscopic photos of double-immunofluorescent staining for TLR4 with α-SMA and for TLR4 with MOMA-2 (a marker for macrophages). (D) VSMC content indicated by intensity of α-SMA staining on day 14 and day 28 (n = 6 per group). (*P<0.05, **P<0.01, n.s. *P*>0.05 compared with NaCl-group. Aortic specimens for double-immunofluorescent staining were obtained 14 days after AAA induction. L indicates lumen. All scale bars represent 50 μm.)

Previous studies have shown that the CaCl_2_ injury may result in experimental AAA in mice within 21 to 42 days [24,25]. In the present study, while the aortic diameter of NaCl-group mice (sham group) exhibited a slight increase across the 28 days (Fig 1A), a significant aortic dilation in CaCl_2_-group mice was found on day 14 and a further dilation on day 28. To characterize the time-course profile of TLR4 protein expression during AAA development, the aortic specimens obtained at different time points following AAA induction were analyzed. The level of TLR4 in the aortic wall of NaCl-group mice did not exhibit remarkable change after AAA induction (Fig 1B). However, TLR4 expression was significantly elevated in CaCl_2_-group mice since day 3, a time point prior to significant aortic dilation on day 14 (Fig 1A). TLR4 expression was persistently upregulated since the initial stage of CaCl_2_-induced AAA formation and reached its peak at 14 days. At 28 days, TLR4 expression was decreased slightly but the level was still higher than that in NaCl-group mice.

The double-immunofluorescent staining for the aortic specimens obtained on day 3, 7 and 14 (i.e., the moment when TLR4 is highly expressed) revealed that TLR4 was predominantly observed in α-smooth muscle actin (α-SMA)-positive VSMCs and to a small extent in macrophage marker (MOMA-2)-positive macrophages (S1 Fig and Fig 1C). Therefore, the decline of TLR4 expression at the late stage may be associated with the quantitative reduction of VSMC content during this interval (Fig 1D). Taken together, these findings revealed that TLR4, derived mainly from VSMCs in the media, is highly abundant since the initial stage of experimental AAA formation in mice.

### TLR4 mediates cytokine and MMP production in VSMCs

**Fig 2:**
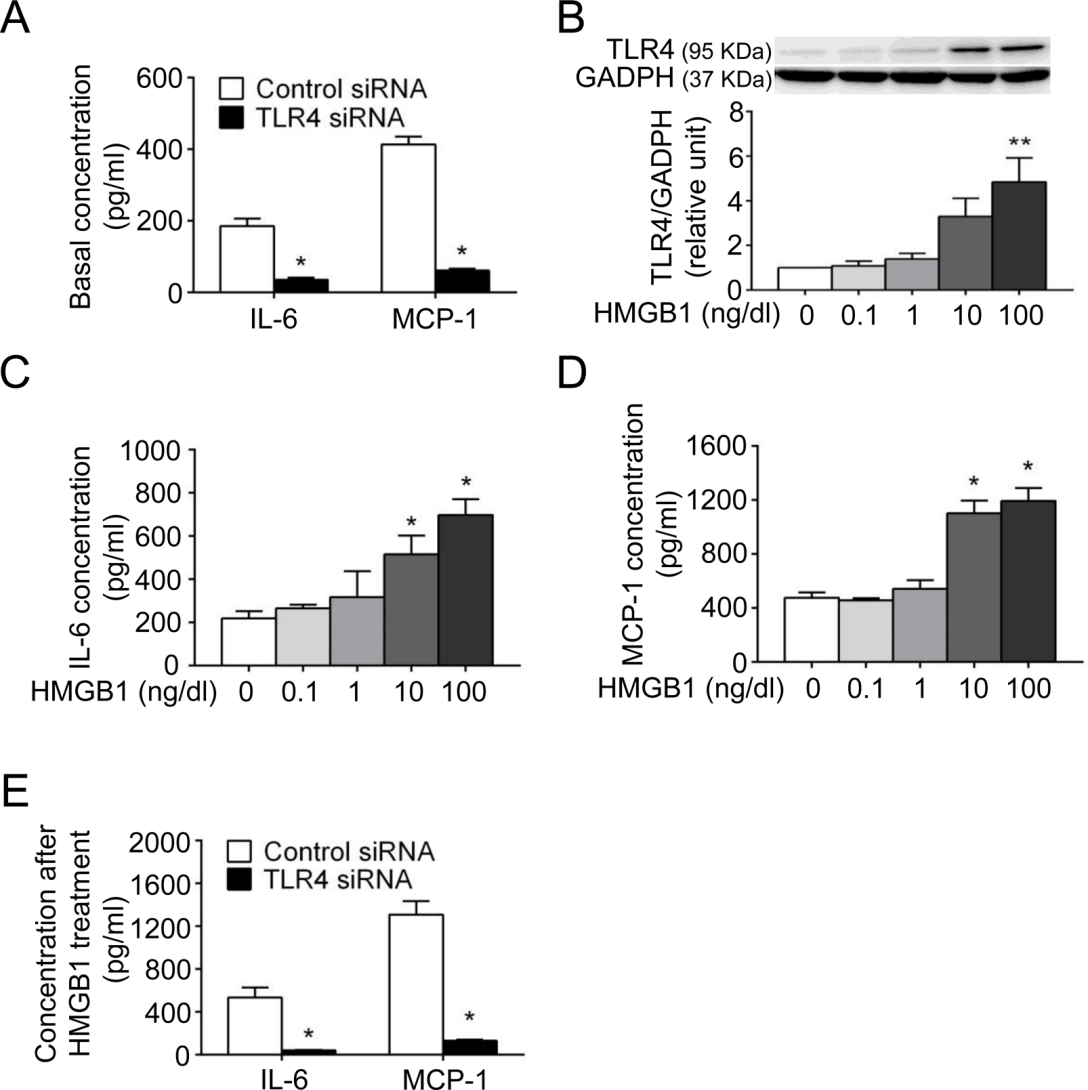
TLR4 mediates cytokine production in HASMCs. (A) Basal levels of IL-6 and MCP-1 were assessed after being cultured in fresh medium without stimulation for 24 hours (n = 4 per group). (B, C, D) The protein expression of TLR4 in cell lysates (B) and levels of IL-6 (C) and MCP-1 (D) in supernatants were assessed after HMGB1 treatment for 24 hours (n = 4 per group). (E) Levels of IL-6 and MCP-1 were assessed after HMGB1 treatment (100 ng/dl) for 24 hours (n = 4 per group). (*P<0.05, **P<0.01 compared with untreated or control siRNA group.)

TLRs are involved in production of the proinflammatory cytokines IL-6 and MCP-1 [27,28], both of which have been associated with macrophage recruitment, chronic inflammation and aneurysm formation [29,30]. IL-6 and MCP-1 can be detected in the normal mouse aorta under an unstimulated condition [27]. Because VSMCs have been one of the primary sources of proinflammatory cytokines in the aortic wall [27,28], human aortic smooth muscle cells (HASMCs) were transfected with TLR4 siRNA to determine whether TLR4 might be involved in basal IL-6 and MCP-1 secretion from HASMCs. After 48-hour transfection, the levels of TLR4 mRNA and protein were suppressed as compared with those in the control siRNA group (S2 Fig). With no ligand stimulation, HASMCs transfected with TLR4 siRNA had reduced IL-6 and MCP-1 secretion compared with those with control siRNA (Fig 2A).

The proinflammatory HMGB1 signaling may sustain either autocrine or paracrine positive feedback loops [8]. HMGB1 is highly abundant in the human AAA and CaCl_2_-induced model and is expressed in inflammatory cells, endothelial cells and also VSMCs [20]. Based on our finding that VSMCs are the major origin of TLR4 expression in AAA, we would therefore evaluate whether TLR4 ligation by HMGB1 might enhance the proinflammatory response (e.g., cytokine release) in VSMCs *in vitro* and whether blockade of TLR4 signaling inhibits the action of HMGB1 on VSMCs. As confirmed by WST-1 assay, recombinant human HMGB1 did not exert cytotoxic effects on HASMCs at the concentrations used (data not shown). Analysis of the cell lysates showed the increase in TLR4 expression corresponding to incremental concentrations of HMGB1 (Fig 2B), suggesting a positive feedback enhancing TLR4 expression on HASMCs after HMGB1 stimulation. In the supernatants, IL-6 (Fig 2C) and MCP-1 (Fig 2D) were enhanced by HMGB1 in a concentration-dependent manner, although TNF-α was not detectable via ELISA. Subsequently, HASMCs were transfected with TLR4 siRNA to determine whether the enhancement of IL-6 and MCP-1 production is mediated by TLR4 signaling. As shown in Fig 2E, siRNA to TLR4 significantly attenuated IL-6 and MCP-1 production, suggesting that HMGB1-enhanced cytokine secretion is at least in part through TLR4 signaling. Taken together, these findings suggested that TLR4 regulates secretion of IL-6 and MCP-1 from VSMCs regardless of ligand stimulation. Activation of TLR4 by HMGB1 may result in amplification of TLR4 signaling and promote release of IL-6 and MCP-1 from VSMCs.

HMGB1 may induce MMP production to promote extracellular matrix (ECM) destruction [12,20]. VSMCs are the main origin of MMP-2 in the aortic wall [31,32]. Thus, we would evaluate whether blockade of TLR4 signaling might inhibit HMGB1-enhanced MMP-2 in VSMCs. In HASMCs, MMP-2 was induced by HMGB1 in a concentration-dependent manner (S3 Fig), and siRNA to TLR4 attenuated MMP-2 production. These results indicated that HMGB1-induced MMP-2 production is mediated at least in part through TLR4.

In summary, blockade of TLR4 signaling by siRNA in VSMCs attenuated HMGB1-enhanced production of proinflammatory cytokines, specifically IL-6 and MCP-1, and matrix-degrading MMP-2, suggesting that TLR4 signaling regulates VSMC inflammation and proteinase production.

### TLR4 deficiency attenuates experimental AAA induced by CaCl_2_ in mice

**Fig 3:**
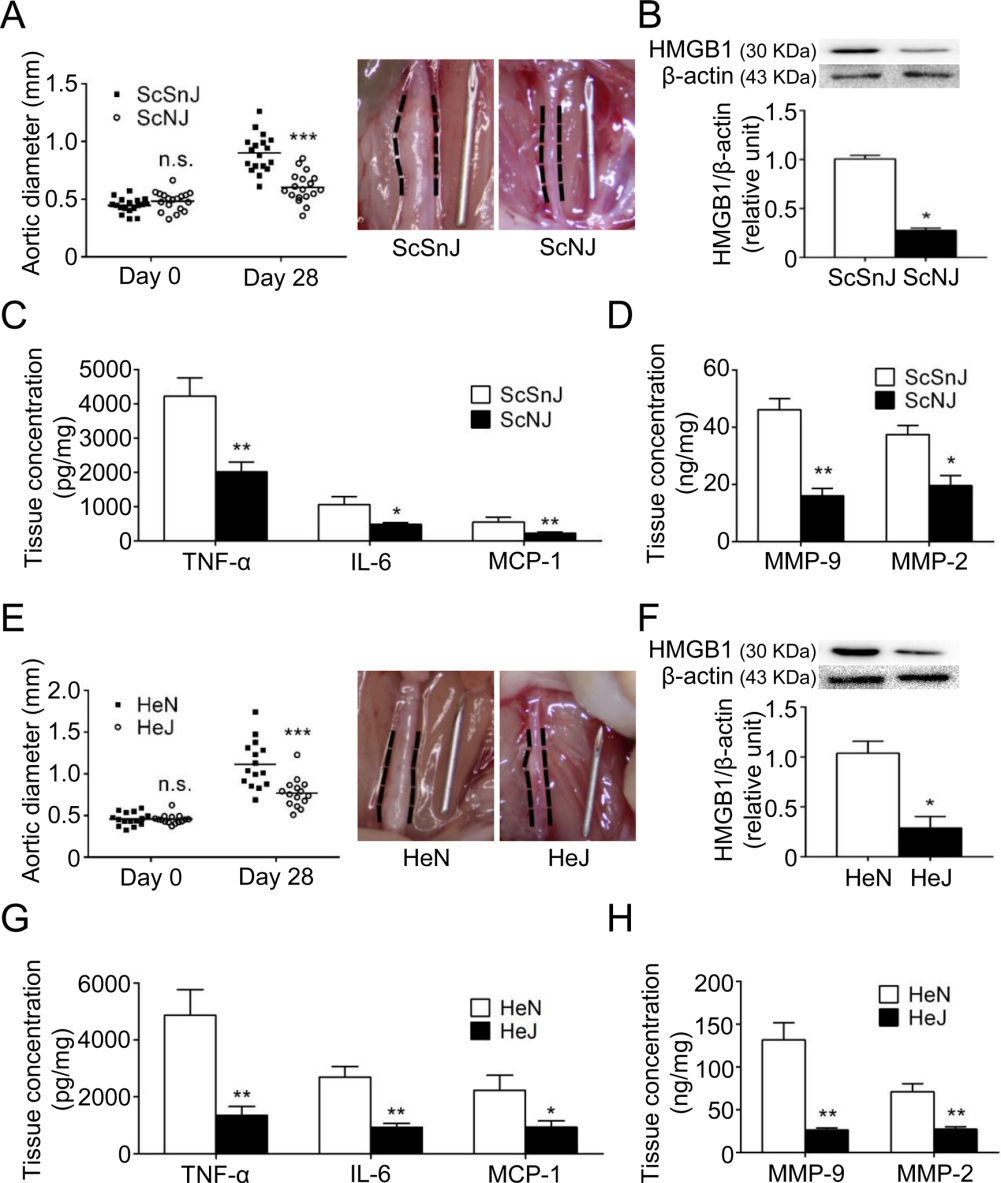
TLR4-knockout (A, B, C, D) and TLR4-mutant (E, F, G, H) mice are resistant to experimental AAA formation. (A, E) Aortic diameter on day 0 and day 28 after AAA induction (n = 18 per group in ScSnJ [wild-type controls] versus ScNJ [TLR4-knockout mice], n = 15 per group in HeN [wild-type controls] versus HeJ [TLR4-mutant mice]). (B, F) HMGB1 level by Western blot (n = 4 per group). (C, G) TNF-α, IL-6, MCP-1 concentrations by ELISA (n = 6 per group). (D, H) MMP levels by ELISA (n = 6 per group). (*P<0.05, **P<0.01, ***P<0.001, n.s. *P*>0.05 compared with respective controls.)

Subsequently we evaluated whether deficiency of TLR4 signaling attenuates CaCl_2_-induced AAA formation *in vivo* using C57BL/10ScNJ mice (ScNJ [TLR4-knockout mice]) and C57BL/10ScSnJ mice (ScSnJ [wild-type controls]). The aortic diameters in ScNJ and ScSnJ mice on day 0 before CaCl_2_ injury were similar (0.48±0.02 mm versus 0.45±0.02 mm, n = 18; *P*>0.05). However, on day 28 after AAA induction, the aortic diameter was significantly smaller in ScNJ mice than in ScSnJ mice (0.60±0.03 mm versus 0.90±0.04 mm; *P*<0.001; Fig 3A). Analysis of the aortic specimens showed that the expression of HMGB1 was significantly lower in ScNJ than in ScSnJ (Fig 3B). The abundance levels of TNF-α, IL-6 and MCP-1 were significantly lower in ScNJ than in ScSnJ (Fig 3C). Compared with ScSnJ, ScNJ had reduced total production of MMP-9 and MMP-2 in the aortic tissue (Fig 3D).

The experimental AAA was also induced in C3H/HeJ mice (HeJ [TLR4-mutant mice]) and C3H/HeN mice (HeN [wild-type controls]). The aortic diameters in HeJ and HeN mice before CaCl_2_ injury were similar (0.46±0.01 mm versus 0.46±0.02 mm, n = 15; *P*>0.05). However, the aortic diameter was significantly smaller in HeJ mice than in HeN mice on day 28 after AAA induction (0.77±0.04 mm versus 1.12±0.07 mm; *P*<0.001; Fig 3E). Consistent with the aortic diameter change, the expression of HMGB1 (Fig 3F) and production of cytokines (Fig 3G) and MMPs (Fig 3H) were reduced in HeJ compared with HeN mice. In summary, CaCl_2_-induced aortic dilatation, vascular inflammation and proteinase production were attenuated in both strains of TLR4-deficient mice.

### Knockout of TLR4 reduces HMGB1 expression, cytokine production and macrophage accumulation during aneurysm initiation

**Fig 4:**
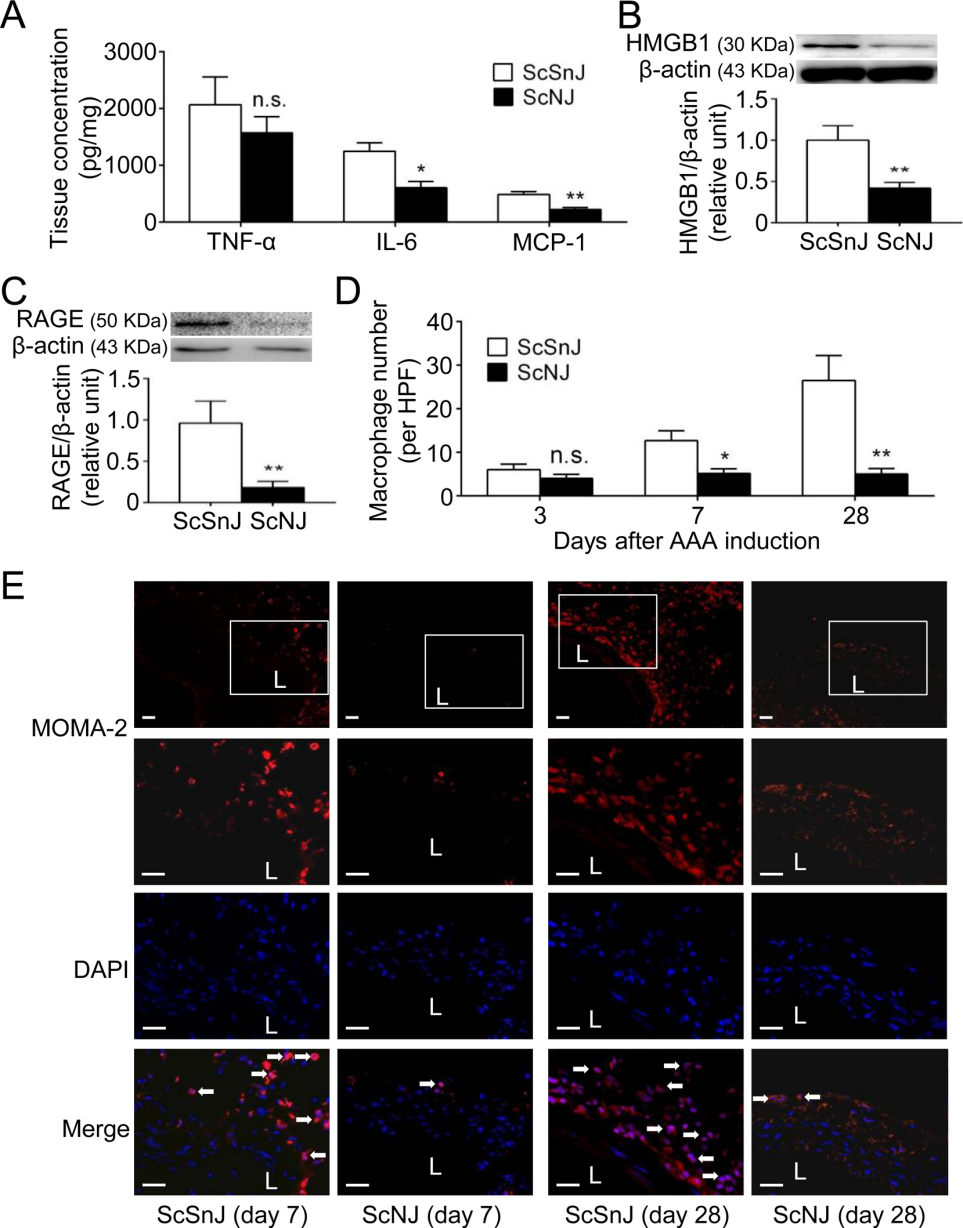
TLR4 knockout reduces cytokine production, HMGB1 and RAGE expression and macrophage accumulation preceding AAA formation. (A) TNF-α, IL-6 and MCP-1 concentration on day 3 by ELISA (n = 6 per group). (B, C) HMGB1 (B) and RAGE (C) levels on day 3 by Western blot (n = 6 per group). (D) Macrophage numbers on day 3, day 7 and day 28 (n = 6 per group). (E) Macrophage immunostaining on day 7 and day 28. (*P<0.05, **P<0.01, n.s. *P*>0.05 compared with ScSnJ mice. L indicates lumen. All scale bars represent 50 μm. White arrows indicate examples of MOMA-2-positive, DAPI-stained macrophages.)

We then induced the experimental AAA in ScNJ and ScSnJ mice to dissect the *in vivo* effects of TLR4 signaling blockade during AAA formation, especially in the early stage. Analysis of the aortic specimens obtained at 3 days revealed that the abundance levels of IL-6 and MCP-1 were significantly lower in ScNJ than in ScSnJ mice (Fig 4A). In addition, HMGB1 expression was significantly attenuated in ScNJ compared with ScSnJ mice (Fig 4B). In ScNJ mice, the protein expression of receptor for advanced glycation end product (RAGE), another HMGB1 receptor that promotes macrophage inflammation and MMP-9 production during aneurysm development [12,33], was also decreased in comparison with ScSnJ mice (Fig 4C). Notably, the differences of TNF-α (Fig 4A) and infiltrating MOMA-2-positive macrophage numbers in ScSnJ and ScNJ mice (6.0±1.3 vs. 4.0±1.0 per high power field [HPF], n = 6 per group; *P*>0.05; Fig 4D) at 3 days were not significant.

Subsequently we examined the histological sections of aortic specimens obtained at 7 days to determine whether the early differences in ScNJ and ScSnJ mice at 3 days would affect the following macrophage infiltration. At 7 days, the infiltrating macrophage number became significantly higher in ScSnJ than in ScNJ mice (12.7±2.3 vs. 5.2±1.1 per HPF, n = 6; *P*<0.05; Fig 4D and 4E). In summary, although the TNF-α level and macrophage number were similar in ScSnJ and ScNJ mice during aneurysm initiation, knockout of TLR4 signaling reduced IL-6 and MCP-1 levels, suppressed HMGB1 and RAGE expression and interfered with consequent accumulation of macrophages.

Finally, we examined the aortic pathologies in ScNJ and ScSnJ mice at 28 days. The MOMA-2-positive macrophages were detected abundantly in ScSnJ mice and significantly fewer macrophages were observed in ScNJ mice (26.5±5.7 vs. 5.0±1.3 per HPF; *P*<0.01; Fig 4D and 4E), compatible with the differences of proinflammatory cytokines and MMP-9 observed in these two groups (Fig 3C and 3D). In parallel with the increased aortic diameter and levels of cytokines and MMPs in ScSnJ mice, histological analysis of the aortic specimens revealed fragmentation of elastic lamina and depletion of VSMCs (S4 Fig). However, elastin integrity and VSMC content in the media layer were highly preserved in ScNJ. Taken together, these findings revealed that knockout of TLR4 ameliorated vascular inflammation preceding AAA formation, thereby protecting mice from aneurysm development.

### TLR4 is located in VSMCs and is upregulated in human AAA

**Fig 5:**
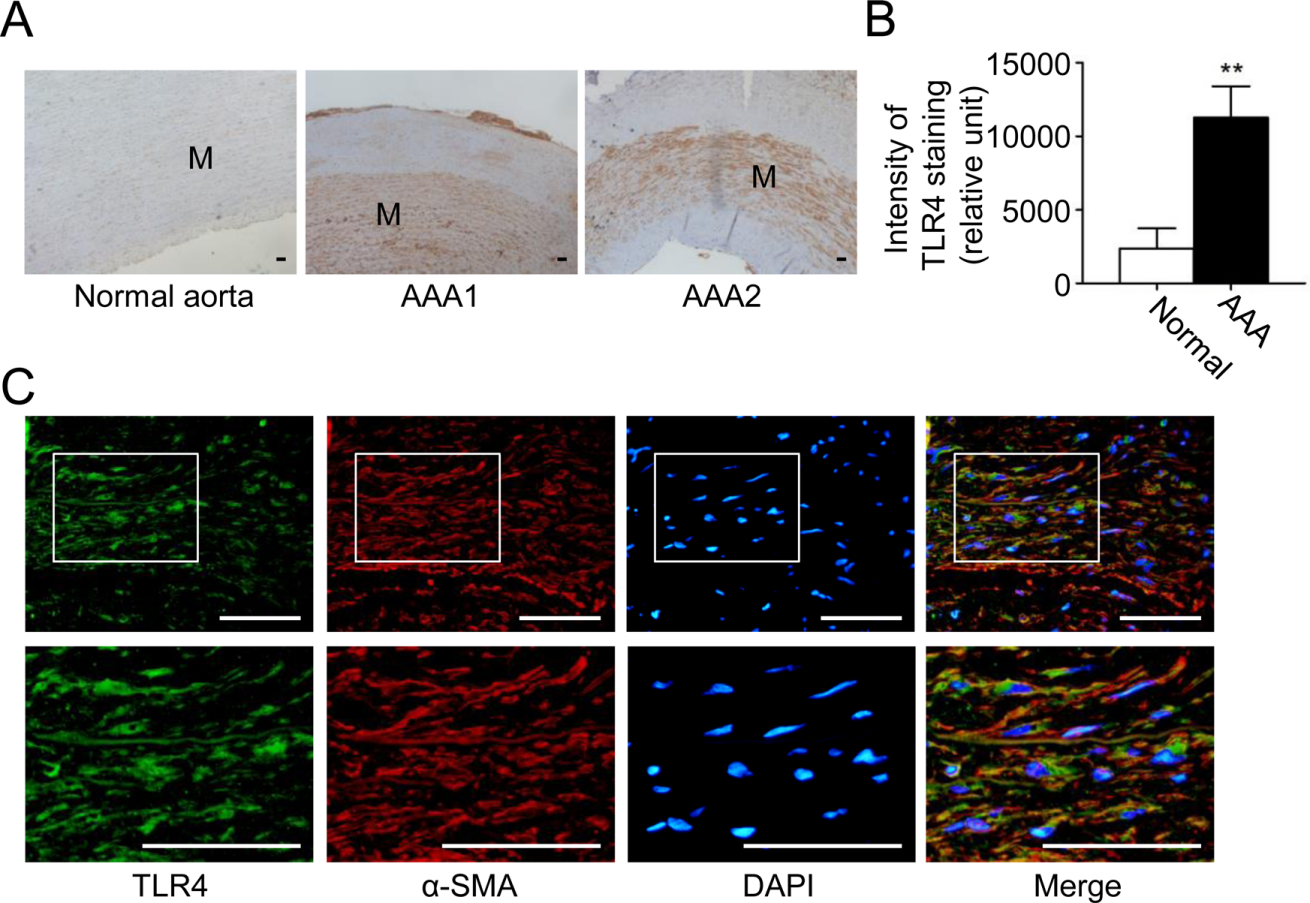
TLR4 is highly expressed in human AAA. (A) Representative microscopic photos of TLR4 expression (stained red-brown) in the human normal aortas and AAAs. (B) TLR4 staining in VSMCs in human normal aortas and AAAs (n = 8 per group). (C) Representative microscopic photos of double-immunofluorescent staining for TLR4 with α-SMA in AAA. (**P<0.01 compared with normal aorta group. M indicates media. Scale bars represent 100 μm [A] and 50 μm [C].)

We have seen that TLR4 signaling is important in experimental mouse AAA. Finally, we sought to determine whether TLR4 might be relevant in human AAA. Immunohistochemically, TLR4 was prevalently expressed in the media layer (Fig 5A) and TLR4 staining was significantly increased in human AAA tissue versus normal aortas (Fig 5B). In addition, the double-immunofluorescent staining showed that TLR4 was mostly observed in α-SMA-positive VSMCs (Fig 5C), and to a lesser extent, in CD68-positive macrophages (S5 Fig), compatible with our findings in the experimental model. Therefore, TLR4 may be relevant in human AAA formation. Together with the observations in experimental AAA in mice, these data suggest that TLR4 might be considered as a novel therapeutic target for pharmacotherapies of AAA.

## Discussion

Studies have investigated the expression and significance of TLR4 in AAA induced by AngII infusion. In apolipoprotein E-knockout mice, the protein expression of TLR4 was dramatically upregulated following AngII infusion for 3 days [34], although TLR4 gene expression was not differentially expressed in mice infused with AngII versus saline at 7 days in another study [35]. TLR4 upregulation was evident particularly in aneurysm-prone supra-renal but not in thoracic and infra-renal aortic regions in this model [34]. Rosiglitazone, a synthetic agonist selective for peroxisome proliferator-activated receptor-γ, effectively suppresses AngII-infusion AAA in part through inhibition of TLR4 signaling [34]. Despite the pronounced attenuation of AngII-infusion AAA formation in mice with whole body TLR4 deficiency, repopulation with TLR4-deficient bone marrow-derived cells in lethally irradiated LDLR-deficient mice exhibited no effects on AAA formation, suggesting that TLR4 exerted its action on AAA through non-hematopoietic cells [22]. While our study was being prepared for publication, a recent study reported that AngII-induced TLR4-mediated AAA in apolipoprotein E-knockout mice is dependent on STAT3 [36]. Using another widely-used CaCl_2_-induced model, our study demonstrated that TLR4 expression was upregulated since the initiation of AAA formation. TLR4 was expressed mainly by VSMCs in the media. TLR4 deficiency, either through deletion of TLR4 gene or an inactivating point mutation in TLR4, attenuated vascular inflammation and development of experimental AAA in mice. Knockout of TLR4 reduced IL-6 and MCP-1 levels and HMGB1 expression, attenuated macrophage accumulation, and eventually suppressed MMP production, elastin destruction and VSMC loss. Histological studies on human specimens revealed that TLR4 was located in VSMCs and was upregulated in AAA versus normal aortas. There is precedence for such an interesting observation that TLR4-bearing tissue-resident cells, rather than hematopoietic cells, contribute to local inflammation [37]. In summary, the results obtained from human samples and different mouse models suggest that upregulation of TLR4 in VSMCs may contribute to AAA formation since aneurysm initiation and corroborate the critical role for TLR4 signaling in AAA.

VSMCs, the major resident cells in the aortic wall, not only maintain aortic architecture by matrix synthesis [31,38] but also participate in the elastolytic process in AAA by proteinase production [18,24]. Recent work began to clarify the proinflammatory mechanisms of VSMCs in aneurysm diseases. VSMC-derived cyclophilin A is required for reactive oxygen species generation, MCP-1 production and MMP-2 activation in AngII-infusion AAA [39]. Due to absence of the human DAMP molecule S100A12 in mice, the VSMC-specific S100A12 transgenic mice are generated to investigate the role of S100A12 in aneurysm formation. These mice exhibit a progressive dilatation of the thoracic aorta in association with increased IL-6 production, activation of transforming growth factor β pathways and enhanced oxidative stress [21]. Recently, an apoptotic mediator protein kinase C-δ mediates VSMC apoptosis and inflammation by stimulating MCP-1 expression in AAA [30]. Also, EP4 prostanoid receptor stimulation increases MMP-2 activity and IL-6 production in VSMCs and EP4 antagonism inhibits the degradation of aortic elastic fiber [40]. More recently, Krüppel-like factor 4 regulates VSMC phenotypic switching and contributes to aneurysm development by enhancing cytokine production [41]. These findings suggest that during aneurysm development, VSMCs may shift toward a proinflammatory state and join directly in the inflammatory process, compatible with the postulate that VSMCs are the soil of AAA development [32].

**Fig 6:**
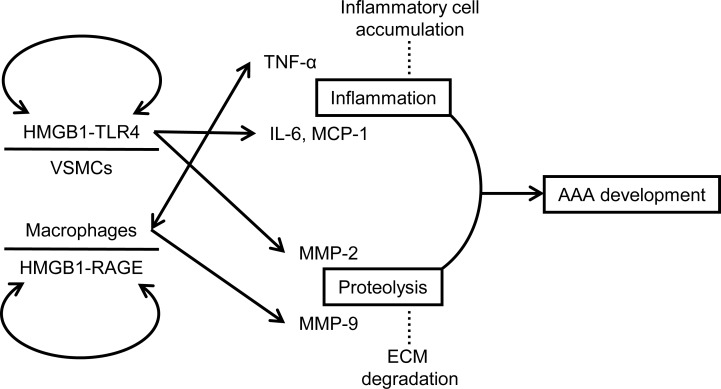
Possible mechanisms of TLR4 in promotion of AAA development. The schematic diagram shows that TLR4 on VSMCs, possibly in conjugation with RAGE on macrophages, sustains both inflammation and proteolysis in the aortic wall during aneurysm development.

In present study, we demonstrate a potential mechanism that TLR4 is an integral signaling molecule in regulating not only proteinase expression but also inflammation in VSMCs during aneurysm formation. TLR4 signaling promotes elastinolysis by mediating MMP-2 production in VSMCs. In addition, TLR4 is essential in maintenance of the basal IL-6 and MCP-1 expression in VSMCs [27], and contributes to a proinflammatory phenotype in VSMCs under stimulation. Consistent with the *in vivo* results showing that TLR4 signaling mediates IL-6 and MCP-1 production in VSMCs, TLR4 deficiency suppressed IL-6 and MCP-1 levels when the macrophage burden remains low in the early stage of AAA. More important, the present study demonstrated that stimulation with HMGB1 enhanced TLR4 expression in VSMCs *in vitro*, and in turn TLR4 deficiency attenuated upregulation of its ligand HMGB1 and another PRR that recognizes HMGB1, RAGE, *in vivo*. Activation of TLR4 signaling by DAMPs may create a positive feedback loop that sustains proinflammatory responses [3]. Our previous work has shown that HMGB1 and RAGE exhibit similar temporal expression patterns during CaCl_2_-induced AAA formation [12]. The present study further demonstrated that despite a late decline possibly due to VSMC loss, TLR4 exhibits a similar trend of upregulation with HMGB1 in the temporal expression pattern since AAA induction. TLR4 and RAGE share common ligands (e.g., HMGB1) and signaling pathways and are both PRRs that may sustain chronic inflammation through NF-κB activation [3,7,8,42]. In addition, S100A12, another DAMP molecule recently recognized as a common ligand of RAGE and TLR4 [19], has been associated with production of IL-6 and MMP-2 and enhancement of aneurysm formation [21]. In conjunction with current knowledge obtained from previous studies [12,20,33], it appears reasonable that both TLR4, mainly on VSMCs, and RAGE, mainly on macrophages, transduce the proinflammatory activity of DAMPs such as HMGB1 to perpetuate inflammatory responses and proteolytic degradation during aneurysm development (Fig 6), as observed in other chronic inflammatory conditions [3,7,8].

Although whole body knockout of TLR4 led to reduction of IL-6 and MCP-1, attenuation of macrophage infiltration and inhibition of ECM degradation and aortic dilatation in mice, the major limitation of our study may be no direct evidence showing that these differences resulted from VSMC-specific TLR4 rather than TLR4 expressed by macrophages. Nevertheless, the majority of TLR4 expression was located to VSMCs both in the human AAA and experimental model. In addition, the *in vivo* observations at the early stage during AAA formation were compatible with the *in vitro* studies on VSMCs. As demonstrated previously, TLR4 exerts its action in AAA through non-hematopoietic cells [22]. These findings suggested that the aortic resident cells such as VSMCs, rather than macrophages, use TLR4 signaling in response to the proinflammatory milieu. Accordingly, we proposed the concept that the proinflammatory status of VSMCs mediated by TLR4 signaling sustains vascular inflammation and leads to AAA formation. Nevertheless, future studies using VSMC-specific TLR4-deficient mice are warranted to validate the findings presented in this study.

In conclusion, our study demonstrates that TLR4, derived mainly from VSMCs, is essential in AAA formation. TLR4 signaling mediates proteinase release from VSMCs, and more important, contributes to AAA formation by promoting cytokine production, specifically IL-6 and MCP-1, during aneurysm initiation and development. These findings support the view that TLR4 regulates the proinflammatory status of VSMCs in AAA. Therefore, targeting TLR4 may serve as a potential therapeutic strategy for AAA, especially in the early stage of aneurysm formation.

## Supporting Information

S1 Fig Representative microscopic photos of double-immunofluorescent staining for TLR4 with α-SMA and for TLR4 with MOMA-2 (a marker for macrophages) on (A) day 3 and (B) day 7. (L indicates lumen. All scale bars represent 50 μm.) (TIF)

S2 Fig Silence of TLR4 in VSMCs using siRNA. VSMCs were transfected with control siRNA or TLR4 siRNA and TLR4 expression was determined by (A) real-time PCR and (B) Western blot analysis 48 hours after transfection. These observations are typical of those obtained in 3 different experiments.
(TIF)

S3 Fig Knockdown of TLR4 attenuates HMGB1-induced MMP-2 in HASMCs. (A) Levels of MMP-2 in supernatants were assessed after HMGB1 treatment for 24 hours (n = 4 per group). (B) Levels of MMP-2 in supernatants were assessed after HMGB1 treatment (100 ng/dl) for 24 hours (n = 4 per group). (*P<0.05, **P<0.01 compared with untreated or control siRNA group.)
(TIF)

S4 Fig TLR4 knockout confers protection against elastin destruction and VSMC loss. (A) Medial elastin degradation on day 28 (n = 6 per group). Elastin degradation grading scales (4 grades) by VVG staining are shown in right panels. (B) VSMC content indicated by intensity of α-SMA staining on day 28 (n = 6 per group). (**P<0.01 compared with ScSnJ mice. L indicates lumen. Blue arrows indicate disrupted elastic lamella. All scale bars represent 50 μm.)
(TIF)

S5 Fig Representative microscopic photos of double-immunofluorescent staining for TLR4 with CD68 in AAA. (All Scale bars represent 50 μm.)
(TIF)

S6 Fig Negative controls of immunostaining. Representative microscopic images of immunostaining with respective isotype-matched IgG controls in (A, B) the mouse CaCl_2_-induced AAA (in comparison with microscopic images shown in Fig 1C and S1 Fig) and (C) the human AAA (in comparison with microscopic images shown in Fig 5C and S5 Fig). (L indicates lumen. All Scale bars represent 50 μm.)
(TIF)

## References

[1] Frantz S, Ertl G, Bauersachs J. Mechanisms of disease: Toll-like receptors in cardiovascular disease. Nat Clin Pract Cardiovasc Med. 2007;4: 444–454. 17653117 10.1038/ncpcardio0938

[2] Kanzler H, Barrat FJ, Hessel EM, Coffman RL. Therapeutic targeting of innate immunity with Toll-like receptor agonists and antagonists. Nat Med. 2007;13: 552–559. 17479101 10.1038/nm1589

[3] Piccinini AM, Midwood KS. DAMPening inflammation by modulating TLR signalling. Mediators Inflamm. 2010;2010 Article ID 672395.: 10.1155/2010/672395 PMC2913853 20706656

[4] Spirig R, Tsui J, Shaw S. The Emerging Role of TLR and Innate Immunity in Cardiovascular Disease. Cardiol Res Pract. 2012;2012: 181394 10.1155/2012/181394 22577589 PMC3346970

[5] Peri F, Piazza M. Therapeutic targeting of innate immunity with Toll-like receptor 4 (TLR4) antagonists. Biotechnol Adv. 2012;30: 251–260. 10.1016/j.biotechadv.2011.05.014 21664961

[6] Tsan MF, Gao B. Endogenous ligands of Toll-like receptors. J Leukoc Biol. 2004;76: 514–519. 15178705 10.1189/jlb.0304127

[7] Ibrahim ZA, Armour CL, Phipps S, Sukkar MB. RAGE and TLRs: relatives, friends or neighbours? Mol Immunol. 2013;56: 739–744. 10.1016/j.molimm.2013.07.008 23954397

[8] van Beijnum JR, Buurman WA, Griffioen AW. Convergence and amplification of toll-like receptor (TLR) and receptor for advanced glycation end products (RAGE) signaling pathways via high mobility group B1 (HMGB1). Angiogenesis. 2008;11: 91–99. 10.1007/s10456-008-9093-5 18264787

[9] Caso JR, Pradillo JM, Hurtado O, Lorenzo P, Moro MA, Lizasoain I. Toll-like receptor 4 is involved in brain damage and inflammation after experimental stroke. Circulation. 2007;115: 1599–1608. 17372179 10.1161/CIRCULATIONAHA.106.603431

[10] Hua F, Ha T, Ma J, Li Y, Kelley J, Gao X, et al Protection against myocardial ischemia/reperfusion injury in TLR4-deficient mice is mediated through a phosphoinositide 3-kinase-dependent mechanism. J Immunol. 2007;178: 7317–7324. 17513782 10.4049/jimmunol.178.11.7317

[11] Qiu J, Xu J, Zheng Y, Wei Y, Zhu X, Lo EH, et al High-mobility group box 1 promotes metalloproteinase-9 upregulation through Toll-like receptor 4 after cerebral ischemia. Stroke. 2010;41: 2077–2082. 10.1161/STROKEAHA.110.590463 20671243 PMC3066477

[12] Lai CH, Shi GY, Lee FT, Kuo CH, Cheng TL, Chang BI, et al Recombinant human thrombomodulin suppresses experimental abdominal aortic aneurysms induced by calcium chloride in mice. Ann Surg. 2013;258: 1103–1110. 10.1097/SLA.0b013e31827df7cb 23295319

[13] Golledge J, Muller J, Daugherty A, Norman P. Abdominal aortic aneurysm: pathogenesis and implications for management. Arterioscler Thromb Vasc Biol. 2006;26: 2605–2613. 16973970 10.1161/01.ATV.0000245819.32762.cb

[14] Miyake T, Morishita R. Pharmacological treatment of abdominal aortic aneurysm. Cardiovasc Res. 2009;83: 436–443. 10.1093/cvr/cvp155 19454489

[15] Baxter BT, Terrin MC, Dalman RL. Medical management of small abdominal aortic aneurysms. Circulation. 2008;117: 1883–1889. 10.1161/CIRCULATIONAHA.107.735274 18391122 PMC4148043

[16] Daugherty A, Cassis LA. Mouse models of abdominal aortic aneurysms. Arterioscler Thromb Vasc Biol. 2004;24: 429–434. 14739119 10.1161/01.ATV.0000118013.72016.ea

[17] Shimizu K, Mitchell RN, Libby P. Inflammation and cellular immune responses in abdominal aortic aneurysms. Arterioscler Thromb Vasc Biol. 2006;26: 987–994. 16497993 10.1161/01.ATV.0000214999.12921.4f

[18] Qin Y, Cao X, Yang Y, Shi GP. Cysteine protease cathepsins and matrix metalloproteinases in the development of abdominal aortic aneurysms. Future Cardiol. 2013;9: 89–103. 10.2217/fca.12.71 23259477 PMC3568657

[19] Foell D, Wittkowski H, Kessel C, Luken A, Weinhage T, Varga G, et al Proinflammatory S100A12 can activate human monocytes via Toll-like receptor 4. Am J Respir Crit Care Med. 2013;187: 1324–1334. 10.1164/rccm.201209-1602OC 23611140

[20] Kohno T, Anzai T, Kaneko H, Sugano Y, Shimizu H, Shimoda M, et al High-mobility group box 1 protein blockade suppresses development of abdominal aortic aneurysm. J Cardiol. 2012;59: 299–306. 10.1016/j.jjcc.2012.01.007 22365948

[21] Hofmann Bowman M, Wilk J, Heydemann A, Kim G, Rehman J, Lodato JA, et al S100A12 mediates aortic wall remodeling and aortic aneurysm. Circ Res. 2010;106: 145–154. 10.1161/CIRCRESAHA.109.209486 19875725 PMC2878187

[22] Owens AP 3rd, Rateri DL, Howatt DA, Moore KJ, Tobias PS, Curtiss LK, et al MyD88 deficiency attenuates angiotensin II-induced abdominal aortic aneurysm formation independent of signaling through Toll-like receptors 2 and 4. Arterioscler Thromb Vasc Biol. 2011;31: 2813–2819. 10.1161/ATVBAHA.111.238642 21960563 PMC3220737

[23] Shang T, Ran F, Qiao Q, Liu Z, Liu CJ. Tanshinone IIA attenuates elastase-induced AAA in rats via inhibition of MyD88-dependent TLR-4 signaling. Vasa. 2014;43: 39–46. 10.1024/0301-1526/a000326 24429329

[24] Longo GM, Xiong W, Greiner TC, Zhao Y, Fiotti N, Baxter BT. Matrix metalloproteinases 2 and 9 work in concert to produce aortic aneurysms. J Clin Invest. 2002;110: 625–632. 12208863 10.1172/JCI15334 PMC151106

[25] Xiong W, MacTaggart J, Knispel R, Worth J, Persidsky Y, Baxter BT. Blocking TNF-alpha attenuates aneurysm formation in a murine model. J Immunol. 2009;183: 2741–2746. 10.4049/jimmunol.0803164 19620291 PMC4028114

[26] Schlederer M, Mueller KM, Haybaeck J, Heider S, Huttary N, Rosner M, et al Reliable quantification of protein expression and cellular localization in histological sections. PLoS One. 2014;9: e100822 10.1371/journal.pone.0100822 25013898 PMC4094387

[27] Song Y, Shen H, Schenten D, Shan P, Lee PJ, Goldstein DR. Aging enhances the basal production of IL-6 and CCL2 in vascular smooth muscle cells. Arterioscler Thromb Vasc Biol. 2012;32: 103–109. 10.1161/ATVBAHA.111.236349 22034510 PMC3241880

[28] Cole JE, Navin TJ, Cross AJ, Goddard ME, Alexopoulou L, Mitra AT, et al Unexpected protective role for Toll-like receptor 3 in the arterial wall. Proc Natl Acad Sci U S A. 2011;108: 2372–2377. 10.1073/pnas.1018515108 21220319 PMC3038746

[29] Tieu BC, Lee C, Sun H, Lejeune W, Recinos A 3rd, Ju X, et al An adventitial IL-6/MCP1 amplification loop accelerates macrophage-mediated vascular inflammation leading to aortic dissection in mice. J Clin Invest. 2009;119: 3637–3651. 10.1172/JCI38308 19920349 PMC2786788

[30] Morgan S, Yamanouchi D, Harberg C, Wang Q, Keller M, Si Y, et al Elevated protein kinase C-delta contributes to aneurysm pathogenesis through stimulation of apoptosis and inflammatory signaling. Arterioscler Thromb Vasc Biol. 2012;32: 2493–2502. 10.1161/ATVBAHA.112.255661 22879584 PMC3442600

[31] Riches K, Angelini TG, Mudhar GS, Kaye J, Clark E, Bailey MA, et al Exploring smooth muscle phenotype and function in a bioreactor model of abdominal aortic aneurysm. J Transl Med. 2013;11: 208 10.1186/1479-5876-11-208 24028184 PMC3847145

[32] Curci JA. Digging in the "soil" of the aorta to understand the growth of abdominal aortic aneurysms. Vascular. 2009;17 Suppl 1: S21–29. 19426606 10.2310/6670.2008.00085 PMC2714584

[33] Zhang F, Kent KC, Yamanouchi D, Zhang Y, Kato K, Tsai S, et al Anti-receptor for advanced glycation end products therapies as novel treatment for abdominal aortic aneurysm. Ann Surg. 2009;250: 416–423. 10.1097/SLA.0b013e3181b41a18 19652591 PMC2921961

[34] Pirianov G, Torsney E, Howe F, Cockerill GW. Rosiglitazone negatively regulates c-Jun N-terminal kinase and toll-like receptor 4 proinflammatory signalling during initiation of experimental aortic aneurysms. Atherosclerosis. 2012;225: 69–75. 10.1016/j.atherosclerosis.2012.07.034 22999334

[35] Spin JM, Hsu M, Azuma J, Tedesco MM, Deng A, Dyer JS, et al Transcriptional profiling and network analysis of the murine angiotensin II-induced abdominal aortic aneurysm. Physiol Genomics. 2011;43: 993–1003. 10.1152/physiolgenomics.00044.2011 21712436 PMC3180735

[36] Qin Z, Bagley J, Sukhova G, Baur WE, Park HJ, Beasley D, et al Angiotensin II-induced TLR4 mediated abdominal aortic aneurysm in apolipoprotein E knockout mice is dependent on STAT3. J Mol Cell Cardiol. 2015;87: 160–170. 10.1016/j.yjmcc.2015.08.014 26299839

[37] Chakravarty S, Herkenham M. Toll-like receptor 4 on nonhematopoietic cells sustains CNS inflammation during endotoxemia, independent of systemic cytokines. J Neurosci. 2005;25: 1788–1796. 15716415 10.1523/JNEUROSCI.4268-04.2005 PMC6725921

[38] Allaire E, Muscatelli-Groux B, Mandet C, Guinault AM, Bruneval P, Desgranges P, et al Paracrine effect of vascular smooth muscle cells in the prevention of aortic aneurysm formation. J Vasc Surg. 2002;36: 1018–1026. 12422114 10.1067/mva.2002.127347

[39] Satoh K, Nigro P, Matoba T, O'Dell MR, Cui Z, Shi X, et al Cyclophilin A enhances vascular oxidative stress and the development of angiotensin II-induced aortic aneurysms. Nat Med. 2009;15: 649–656. 10.1038/nm.1958 19430489 PMC2704983

[40] Yokoyama U, Ishiwata R, Jin MH, Kato Y, Suzuki O, Jin H, et al Inhibition of EP4 signaling attenuates aortic aneurysm formation. PLoS One. 2012;7: e36724 10.1371/journal.pone.0036724 22570740 PMC3343028

[41] Salmon M, Johnston WF, Woo A, Pope NH, Su G, Upchurch GR Jr., et al KLF4 regulates abdominal aortic aneurysm morphology and deletion attenuates aneurysm formation. Circulation. 2013;128: S163–174. 10.1161/CIRCULATIONAHA.112.000238 24030402 PMC3922284

[42] Schmidt AM, Yan SD, Yan SF, Stern DM. The multiligand receptor RAGE as a progression factor amplifying immune and inflammatory responses. J Clin Invest. 2001;108: 949–955. 11581294 10.1172/JCI14002 PMC200958

