# Supplementary figures and images for: Toll-Like Receptor 4 Is Essential in the Development of Abdominal Aortic Aneurysm
Source: PLoS One. 2016 Jan 7;11(1):e0146565. doi: 10.1371/journal.pone.0146565 (PMC4711799; doi:10.1371/journal.pone.0146565)

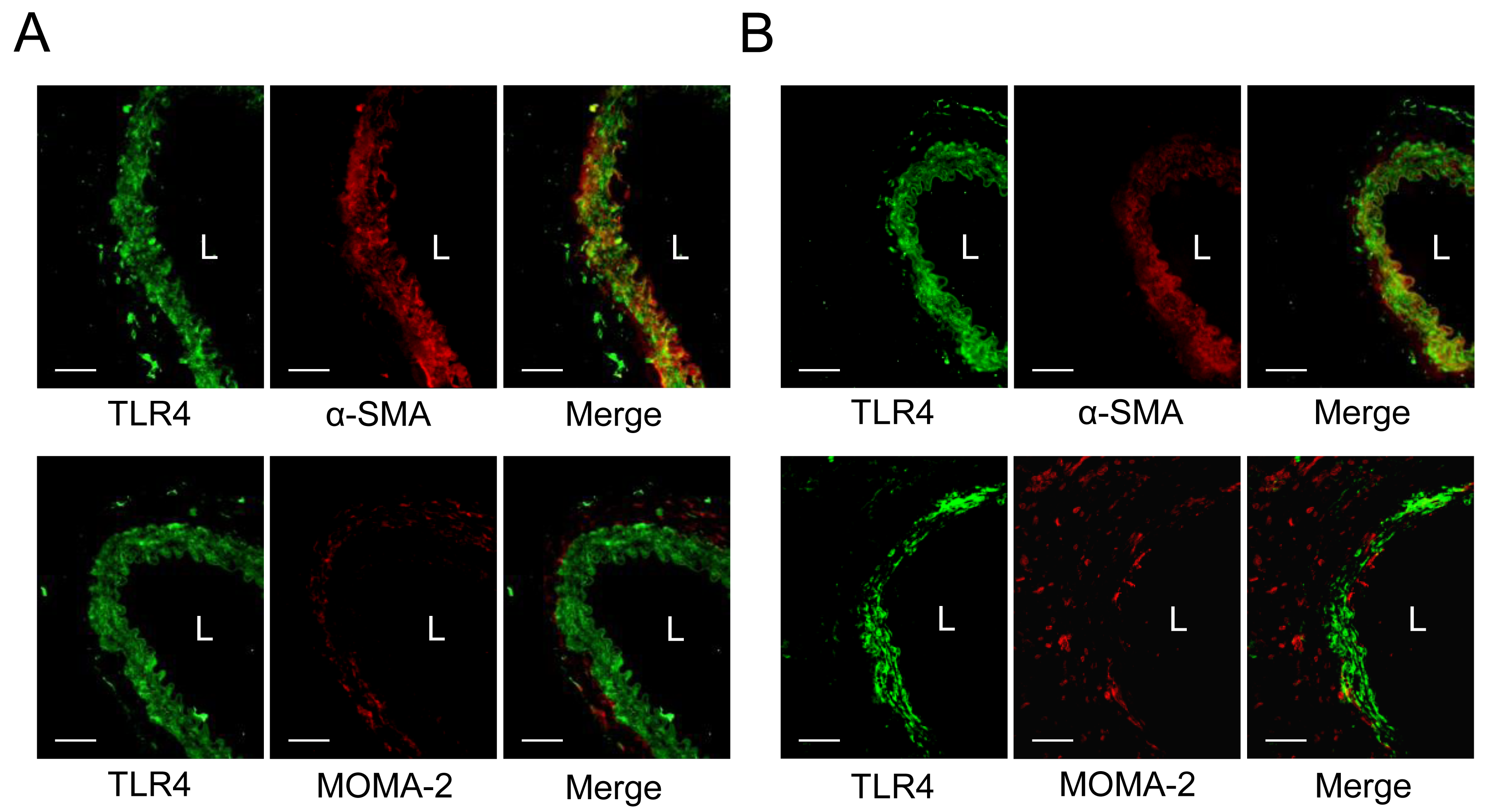

Supplement: S1 Fig — (TIF) [file pone.0146565.s001.tif]

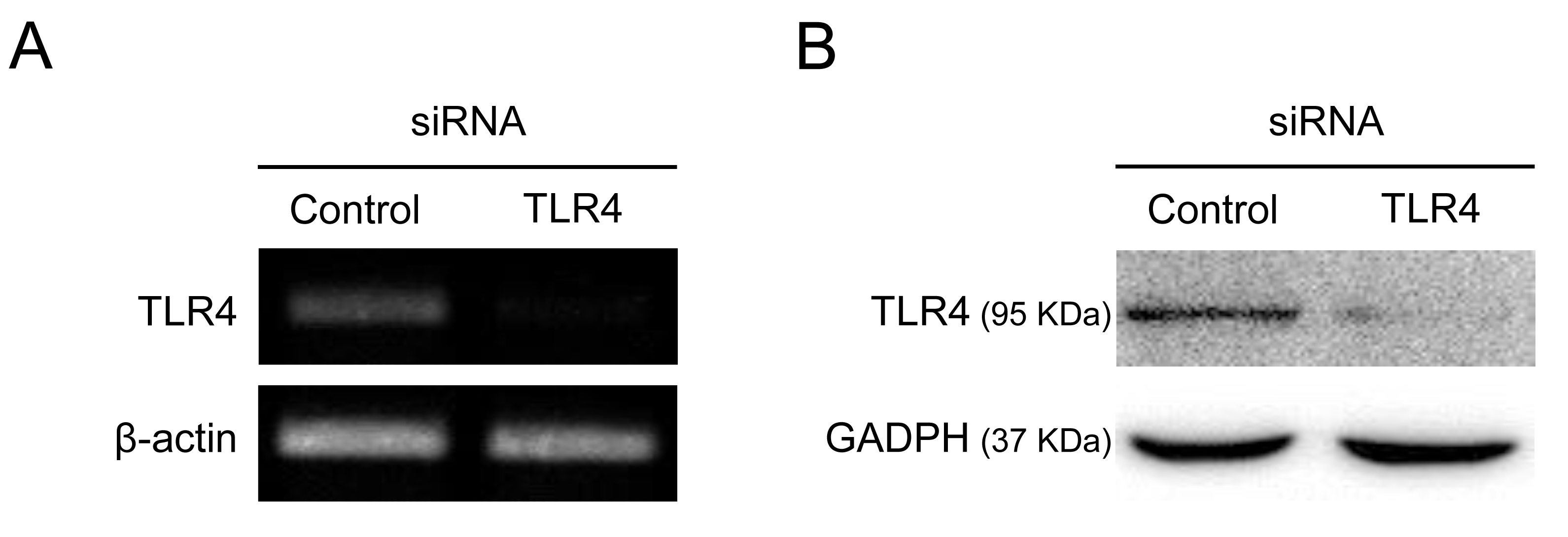

Supplement: S2 Fig — VSMCs were transfected with control siRNA or TLR4 siRNA and TLR4 expression was determined by (A) real-time PCR and (B) Western blot analysis 48 hours after transfection. These observations are typical of those obtained in 3 different experiments. (TIF) [file pone.0146565.s002.tif]

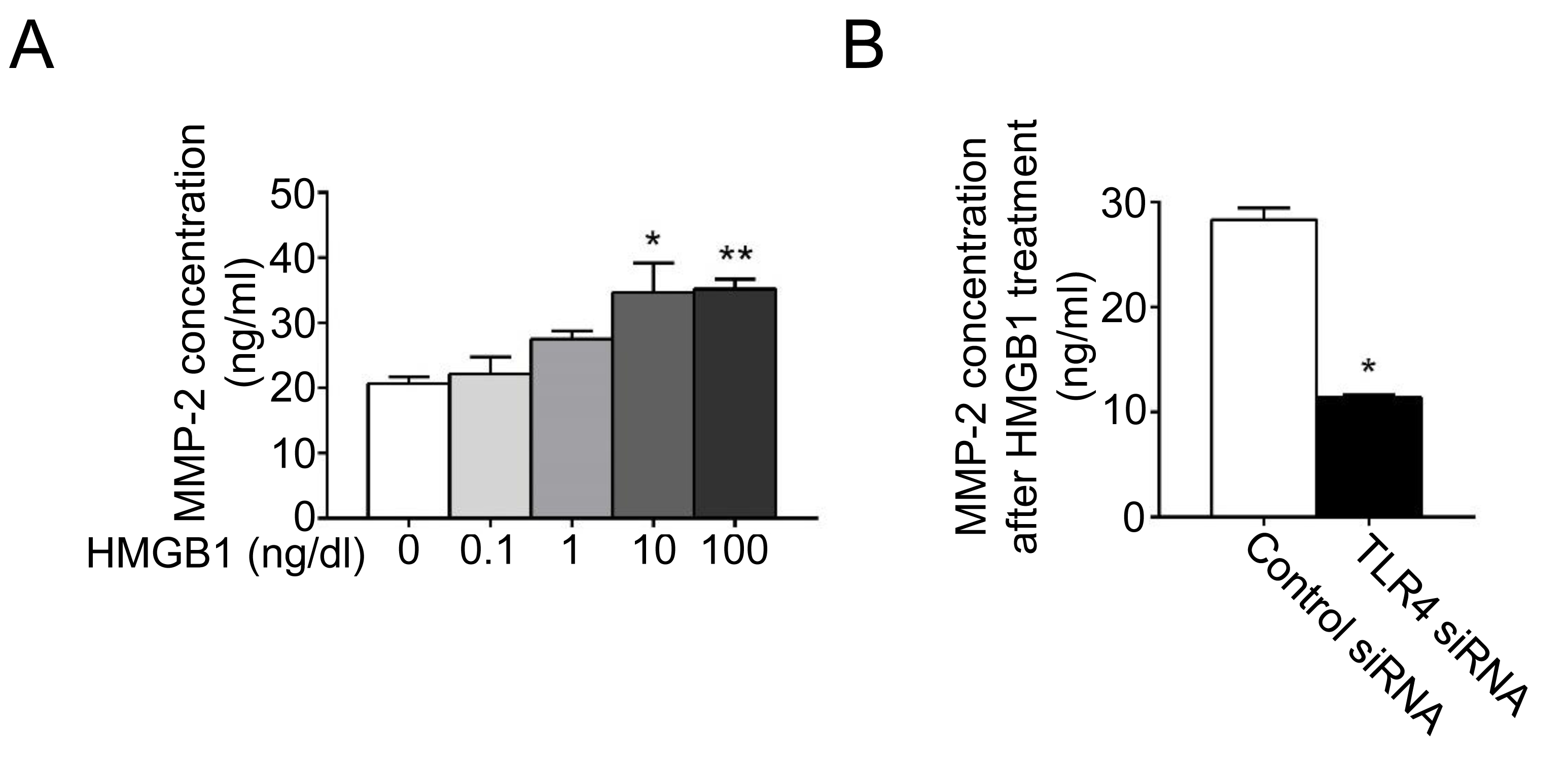

Supplement: S3 Fig — (A) Levels of MMP-2 in supernatants were assessed after HMGB1 treatment for 24 hours (n = 4 per group). (B) Levels of MMP-2 in supernatants were assessed after HMGB1 treatment (100 ng/dl) for 24 hours (n = 4 per group). (*P<0.05, **P<0.01 compared with untreated or control siRNA group.) (TIF) [file pone.0146565.s003.tif]

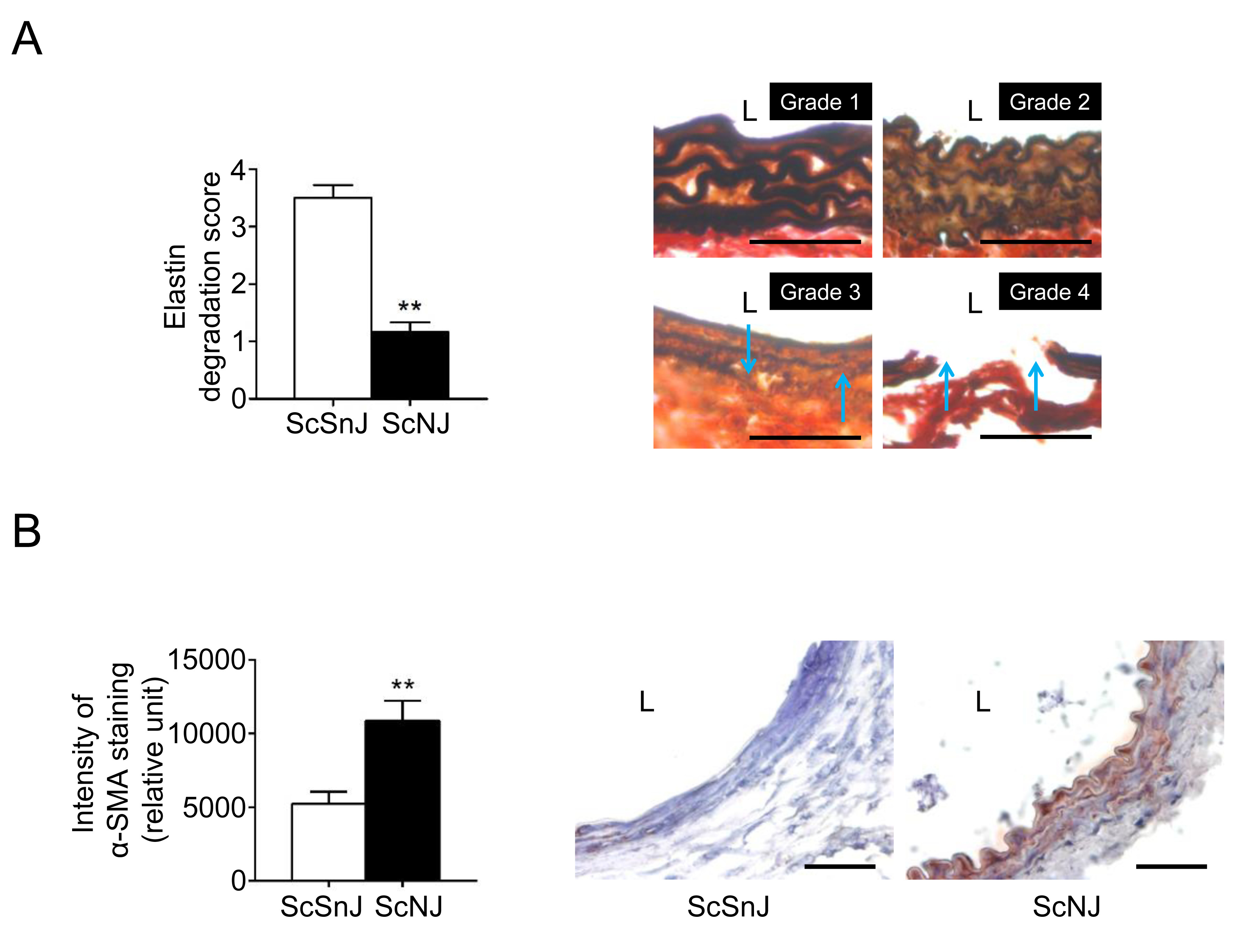

Supplement: S4 Fig — (A) Medial elastin degradation on day 28 (n = 6 per group). Elastin degradation grading scales (4 grades) by VVG staining are shown in right panels. (B) VSMC content indicated by intensity of α-SMA staining on day 28 (n = 6 per group). (**P<0.01 compared with ScSnJ mice. L indicates lumen. Blue arrows indicate disrupted elastic lamella. All scale bars represent 50 μm.) (TIF) [file pone.0146565.s004.tif]

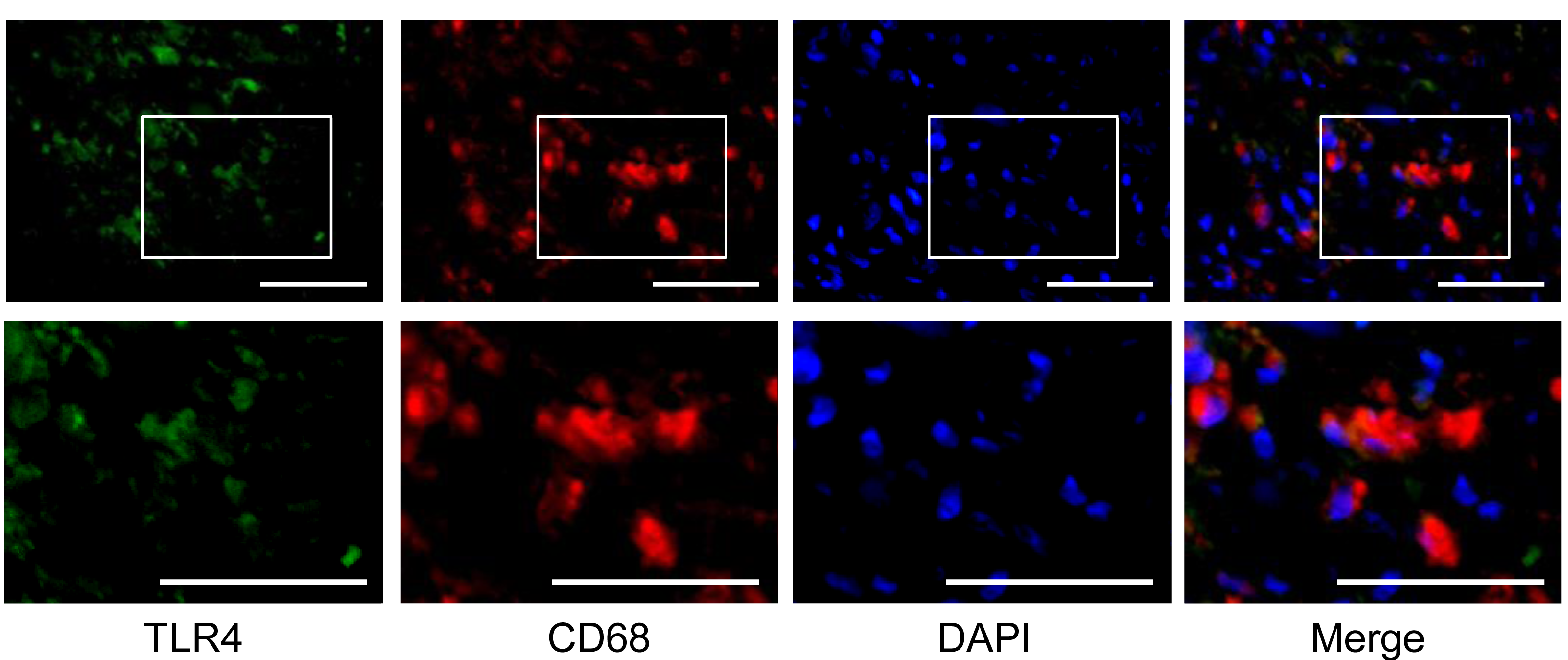

Supplement: S5 Fig — (All Scale bars represent 50 μm.) (TIF) [file pone.0146565.s005.tif]

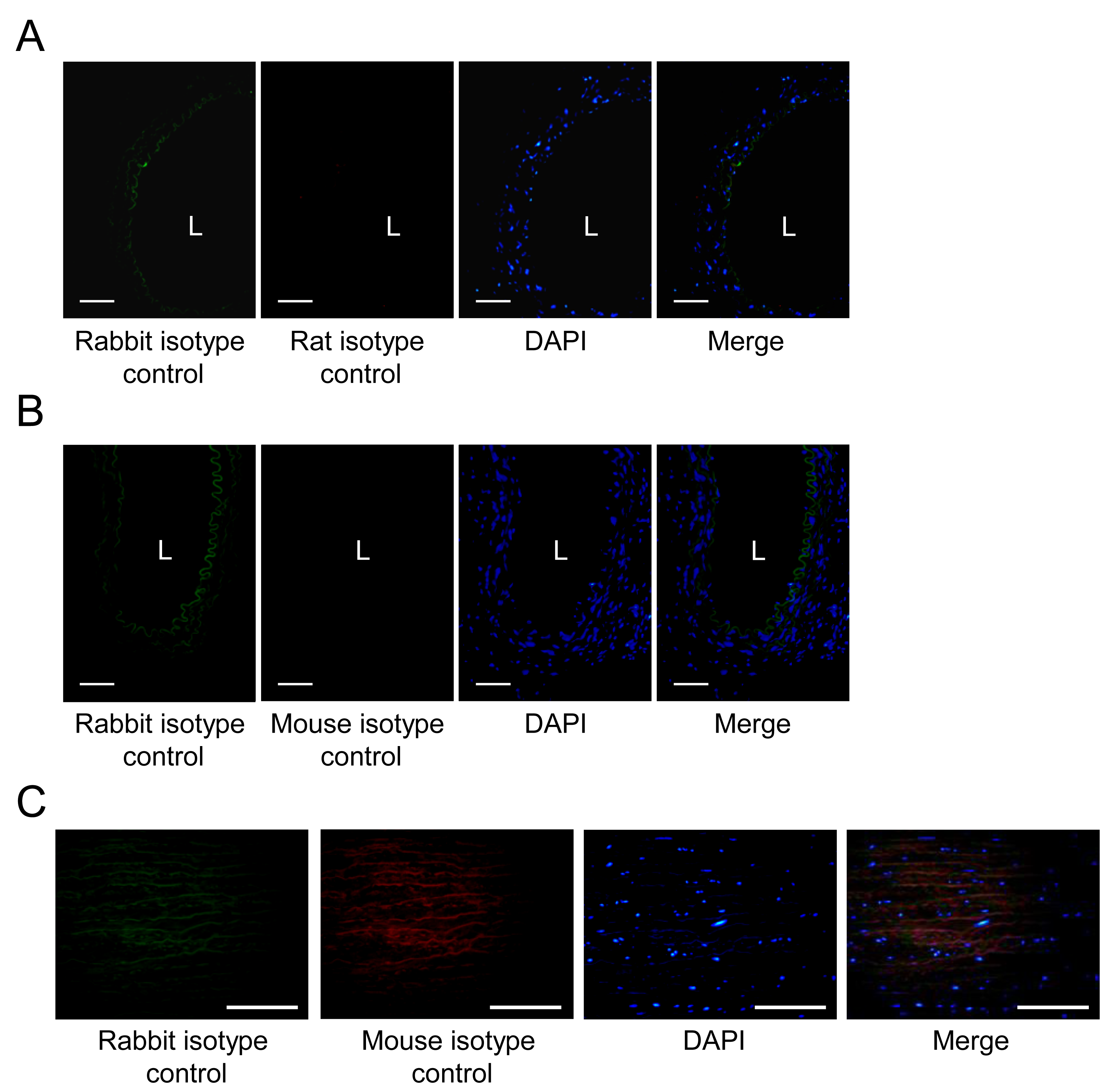

Supplement: S6 Fig — Representative microscopic images of immunostaining with respective isotype-matched IgG controls in (A, B) the mouse CaCl2-induced AAA (in comparison with microscopic images shown in Fig 1C and S1 Fig) and (C) the human AAA (in comparison with microscopic images shown in Fig 5C and S5 Fig). (L indicates lumen. All Scale bars represent 50 μm.) (TIF) [file pone.0146565.s006.tif]
